# Investigation of Endocytic Pathways for the Internalization of Exosome-Associated Oligomeric Alpha-Synuclein

**DOI:** 10.3389/fnins.2017.00172

**Published:** 2017-03-30

**Authors:** Marion Delenclos, Teodora Trendafilova, Divya Mahesh, Ann M. Baine, Simon Moussaud, Irene K. Yan, Tushar Patel, Pamela J. McLean

**Affiliations:** ^1^Department of Neuroscience, Mayo ClinicJacksonville, FL, USA; ^2^Departments of Transplantation and Cancer Biology, Mayo ClinicJacksonville, FL, USA; ^3^Mayo Clinic Graduate School of Biomedical Sciences, Mayo ClinicJacksonville, FL, USA

**Keywords:** αsyn oligomers, cellular uptake, extra cellular vesicles, exosomes, endocytosis

## Abstract

Misfolding and aggregation of alpha-synuclein (αsyn) resulting in cytotoxicity is a hallmark of Parkinson's disease (PD) and related synucleinopathies. The recent body of evidence indicates that αsyn can be released from neuronal cells by nonconventional exocytosis involving extracellular vesicles (EVs) such as exosomes. The transfer of αsyn between cells has been proposed to be an important mechanism of disease propagation in PD. To date, exosome trafficking mechanisms, including release and cell-cell transmission, have not been fully described. To gain insight into the mechanisms involved, exosomes were purified from conditioned media of stable cells secreting αsyn oligomers. A novel bimolecular protein complementation assay was used to detect exosomes containing αsyn oligomers. Recipient cells were treated with exosomes containing αsyn oligomers or “free” non-exosome-associated αsyn oligomers and internalization was monitored. We demonstrate that cell-derived exosome-associated αsyn oligomers can be efficiently internalized by recipient cells. Interestingly exosome-free αsyn oligomers isolated from conditioned medium were not internalized but remained bound to the extracellular surface. To investigate the endocytic pathway(s) required for the exosome uptake different pharmacological inhibitors of caveolin-dependent, clathrin-dependent, and macropinocytosis pathways were utilized. Surprisingly, none of these pathways appear to play a significant role in the internalization of exosome-associated αsyn oligomers. Finally, the role of heparin sulfate proteoglycans (HSPGs) in exosome-associated αsyn internalization was investigated using genetic approach. Despite previous studies showing HSPGs can modulate internalization of fibrillar αsyn, genetic manipulations did not attenuate internalization of exosome-associated αsyn oligomers in our hands, suggesting that exosome-associated αsyn is internalized via an alternative endocytic pathway(s) that has yet to be elucidated.

## Introduction

Parkinson's disease (PD) is the second most common neurodegenerative disorder, with only partial symptomatic therapy and no disease modifying therapies. The accumulation and aggregation of the protein alpha-synuclein (αsyn) is causatively linked both genetically and pathologically to PD (Polymeropoulos et al., 1997; Spillantini et al., 1997). αSyn is a small presynaptic protein generally found under physiological conditions as a monomeric, unfolded, and soluble protein. However, several factors can trigger αsyn misfolding leading to the formation of intermediate soluble species, oligomers, and protofibrils, which become insoluble β-sheet-rich fibrils and are found deposited into the cytosol of affected neurons and are known as Lewy bodies (Forno, 1996; Spillantini et al., 1997; Halliday et al., 2011). A large body of evidence points to the prefibrillar αsyn oligomers as a source of αsyn-induced toxicity (Conway et al., 2000; Volles et al., 2001; Lashuel et al., 2002; Sharon et al., 2003; Kayed et al., 2004; Danzer et al., 2007, 2011; Winner et al., 2011) and although the exact mechanism of αsyn-induced toxicity remains unknown, inhibition of αsyn oligomerization has been explored as a potential therapeutic strategy and explains the growing interest in monitoring these species. Another feature of PD is the possible spread of misfolded αsyn along distinct neuroanatomical pathways suggesting a sequential transfer and misfolding event underlies the pathology and progression of the disease. The propagation of aggregated proteins between cells has been proposed to be an important mechanism of disease propagation not only in PD (El-Agnaf et al., 2006; Desplats et al., 2009; Olanow and Prusiner, 2009) but also other neurodegenerative disorders such as Alzheimer's disease, Amyotrophic lateral sclerosis (ALS), or Huntington's disease (Ren et al., 2009; Münch et al., 2011; Guo and Lee, 2014). The process by which oligomers are internalized into target cells and exit cells remains largely unknown and has resulted in great debate in the literature. The involvement of extracellular vesicles (EVs) such as exosomes has received increasing attention in synucleinopathies and in the field of neurodegeneration in general (Ghidoni et al., 2008; De Toro et al., 2015; Quek and Hill, 2016). EVs can function as shuttles for the delivery of cargo between cells and play a crucial role in cell-to-cell communication. Interestingly, in the last decade a number of proteins involved in neurodegenerative disorders have been identified in their normal and pathogenic states to be associated with EVs and in particular exosomes including PrP, tau, Aβ, SOD1, and most importantly αsyn (Rajendran et al., 2006; Gomes et al., 2007; Vella et al., 2007; Danzer et al., 2012; Saman et al., 2012). This discovery underscores a potential pathogenic role for EVs as vehicles to transfer toxic aggregated proteins between cells in disease conditions.

Exosomes are small membrane vesicles ranging from 40 to 100 nm in diameter that represent one population of EVs. Various cell types including neurons, astrocytes, and glia, have the capacity to release these vesicles into the extracellular space where they are later found in body fluids such as urine, blood, and cerebrospinal fluid (CSF; Théry et al., 2006; Keller et al., 2011). Exosomes originate from the inward budding of endosomes into multivesicular bodies (MVBs) to form intraluminal vesicles that are then directed outside of the cells (Kowal et al., 2014). They can carry lipids, proteins, and nucleic acids that are eventually released into neighboring cells cytoplasm. The process of internalization is still elusive but can occur through direct fusion of the plasma membrane or required receptor-mediated endocytosis. Depending on the recipient cells, internalization has been described to occur through various mechanisms such as clathrin-mediated endocytosis in neuronal cells (Frühbeis et al., 2013; Tian et al., 2014), clathrin-independent but cholesterol- and lipid raft-dependent endocytosis in endothelial and some tumor cells (Svensson et al., 2013) or caveolae-dependent endocytosis in epithelial cells (Nanbo et al., 2013).

EVs seem to play an important role in PD transmission and consequently have generated great interest for potential applications in both diagnostics and therapeutics of neurodegenerative disorders. Thus, it appears crucial to better understand their molecular mechanisms of interaction with target cells. The objective of the present study is to elucidate the role of exosomes in the transmission of oligomeric forms of αsyn and shed new light on the route(s) leading to their internalization into recipient cells. We previously described a cell based assay where exosome–associated αsyn oligomers could be produced and monitored using a highly sensitive bioluminescence protein complementation assay (Danzer et al., 2012). Taking advantage of this new technology, we purified exosomes from conditioned media of a newly generated stable cells line (Moussaud et al., 2015) secreting αsyn oligomers and investigated endocytic pathway(s) required for their uptake. An understanding of these events will clarify the therapeutic potential of enzymes that regulate protein trafficking and degradation in synucleinopathies.

## Materials and methods

### Cells culture

A stable human H4 neuroglioma cell line coexpressing human αsyn tagged with either the amino-terminal (SL1) or the carboxy-terminal fragments (SL2) of *Gaussia princeps* luciferase was generated and described previously (Moussaud et al., 2015). SL1SL2 cells were maintained at 37°C in a 95% air/5% CO_2_ humidified incubator in Opti-MEM supplemented with 10% FBS. To block the expression of the transgenes (SL1 and SL2), cells were cultured in the presence of 1 μg/ml tetracycline (Invitrogen). Human H4 neuroglioma cells (H4 cells; ATCC, USA) were maintained in Opti-MEM I Reduced Serum Medium (Life Technologies) supplemented with 10% Fetal Bovine Serum (Sigma) (FBS) at 37°C in a 95% air/5% CO_2_ humidified incubator. Cells were plated 24 h prior to uptake assay growing to 80–90% confluency. Chinese hamster ovary (CHO) cells, Wild-type (CHO-K1) and mutant (pgsA-745, pgsD-677) (ATCC, USA) were maintained at 37°C in a 95% air/5% CO_2_ humidified incubator and were grown in Opti-MEM supplemented with 10% FBS.

### Exosome isolation purification

Secreted extracellular vesicles were isolated from cell culture medium of SL1SL2 stable cell line by multiple centrifugation steps essentially as previously described (Danzer et al., 2012). Sub confluent SL1SL2 cells were cultured in FBS-free OptiMEM without phenol red (Thermo Fisher). Conditioned medium was collected after 96 h and centrifuged at 300 × g for 10 min to remove cell debris. This was followed by two filtration steps with 45 and 22 uM filtration systems (Fisher Scientific) and then a 10,000 × g centrifugation at 4°C for 30 min. Exosomes were pelleted by ultracentrifugation at 100,000 × g for 70 min repeated twice. To validate the presence and purity of intact exosomes, western blot analysis was performed and the size of the vesicles was analyzed using nanoparticle tracking system, the NanoSight LM10 (Malvern, Amesbury, UK) and NTA2.3 software. Each vesicle preparation was stored at −80°C until further use.

### Cellular uptake assay

For internalization assay, H4 cells were grown to subconfluency on a 96 well plate and incubated with SL1SL2 exosomes (3 × 10^8^ particles/mL) diluted in phenol red-free and serum-free conditions for the indicated times at 37°C, washed twice for 5 min each with PBS and incubated for 1 min with 0.01% trypsin to remove any bound protein on the external cell surface when indicated. Each of the samples was analyzed for internalization by performing a luciferase assay. Luciferase activity from oligomer formation was measured in live cells using a Wallac Victor 3 multilabel counter (PerkinElmer; Waltham, MA) at 480 nm following the injection of the cell permeable substrate, coelenterazine (20 mM, NanoLight). Uptake assays were also performed in the presence of pharmacological compounds and analyzed for luminescence after 1 h at 37 or 4°C when indicated.

### Pharmacological treatments

H4 cells were preincubated for 30 min to 2 h at 37°C in DMEM containing different inhibitors of endocytosis. For inhibition of clathrin-dependent endocytosis, samples were pretreated for 30 min with 10 mg/ml Chlorpromazine (CPZ); for disruption of caveolar endocytosis, cells were pretreated for 2 h with 25 mg/ml nystatin and lastly cells were pretreated with cytochalasin D at 2 μM to block macropinocytosis. All reagents and compounds were from Sigma-Aldrich (St. Louis, MO, USA) unless otherwise noted. Inhibitors were present in all subsequent steps of the experiments. The specificity of each inhibitor treatment was evaluated by monitoring the internalization of fluorescent endocytic markers: Alexa-488-Transferrin (Tfn), Alexa-488-Dextran D, and Alexa-488-Choleratoxin B. Cell viability was 90% for each inhibitor treatment as judged by Trypan blue staining.

### Western blotting

Cells or exosomes were washed with ice-cold PBS and lysed in a reducing RIPA buffer (Millipore) supplemented with 5% (v/v) complete mini protease inhibitor mixture (Roche Diagnostics). For CD9 antibody requiring non-reduced conditions, samples were lysed in Triton-X buffer [20 mM Tris-HCl, pH 8.0, 137 mM NaCl, 1% (v/v) Triton X-100, 2 mM EDTA] supplemented with complete mini protease inhibitor mixture. Proteins were then separated by electrophoresis in a 4–12% Bis-Tris gradient gels, blotted on PVDF membranes (Millipore), and developed using HRP substrate. Immunoblots were probed with the following antibodies for 1 h at room temperature: Flotillin-1 (1:3,000, rabbit polyclonal, Novus), TSG101 (1:1000, rabbit polyclonal, Abcam), CD9 (1:1000, mouse monoclonal, Novus), GM130 (1:5000, rabbit polyclonal, Abcam), αsyn (anti-αsyn clone 4B12, 1:3,000, mouse monoclonal, Covance), and Actin (anti-β-actin 1:10,000, rabbit polyclonal, Sigma). The membranes were washed and incubated with HRP-conjugated secondary antibodies (Southern BioTech) for 1 h at room temperature. Protein was detected by using ECL Western Blotting substrate (Millipore) and a chemiluminescence camera.

### Statistic

All quantified data represent an average of triplicates. Data were analyzed using GraphPad Prism 6 (San Diego, CA) and are presented as mean standard ± standard error of the mean (S.E.M.) Statistical significance was determined using a Student's *t*-test or One-way analysis of variance with Tukey's multiple comparison *post-hoc*. *p* < 0.05 was considered significant.

## Results

### SL1SL2 cell line produced exosomes-associated αsyn oligomers

**Figure 1:**
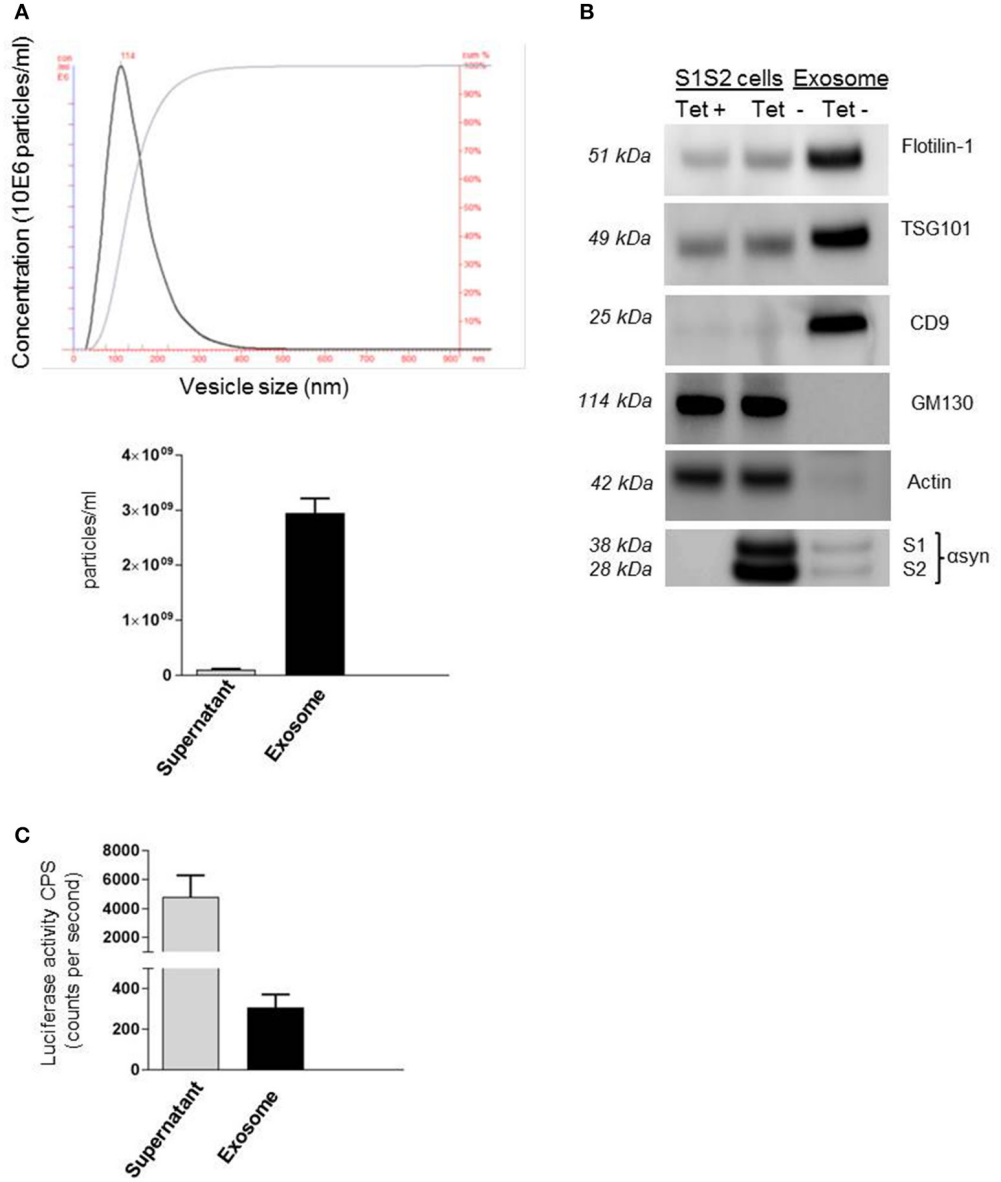
**Detection of αsyn oligomers in SL1 SL2 human neurogliomal cell derived -exosome. (A)** Characterization of H4-derived vesicles by nanoparticle tracking; (Top) Representative particle size distribution of exosome preparation; (bottom) Average concentration of nano vesicles in the exosomal fraction as well as in the supernatant. **(B)** Immunoblot analysis of SL1SL2 cells and exosome-like vesicles for the exosomal markers flotillin-1, CD9, TSG101, the ER marker GM130, and Actin. αSyn was detected with an antibody speficic for human αsyn (clone 4B12) in the cell line and in the exosome **(C)** αsyn oligomers detection via luciferase assay in the supernatant and exosomes isolated from Sl1 to Sl2 cell line.

Recent work from our group and others (Emmanouilidou et al., 2010; Alvarez-Erviti et al., 2011; Danzer et al., 2012) suggests that secretion of αsyn, and especially oligomeric species, is in association with membrane vesicles, identified as exosomes. These carriers of αsyn can be transported from cell to cell and thus may provide an explanation for the spread of αsyn pathology in PD patients. Despite growing evidence for the role of these extracellular vesicles as carriers of αsyn, little is known about the mechanism of entry into neighboring cells. In this present study we took advantage of a recently generated stable cell line (Moussaud et al., 2015) to purify exosomes containing αsyn oligomers and investigate internalization into H4 neuroglioma recipient cells. Briefly, in this SL1SL2 cell line, two h αsyn proteins fused to N- or C-terminal halves of a luciferase reporter can reconstitute the enzymatic activity of gaussia luciferase when αsyn-αsyn interactions occur, thus providing a readout for the presence of αsyn oligomeric species. The culture medium of SL1SL2 stable cells was collected and sequentially centrifuged in order to isolate EVs. The resulting exosome pellet first underwent quality control analyses. The homogeneity of the exosomes used in this study was confirmed by nanoparticle tracking analysis showing a population of vesicles with a size distribution peaking at a diameter between 90 and 120 nm (Figure 1A). In this study, the supernatant obtained after the first ultracentrifuge step was kept and also analyzed by Nanosight. As presented in Figure 1A (bottom), the amount of exosomes remaining in the supernatant (1.02 × 10^8^ particles/ml) is negligible compared to the much higher vesicle number in the exosome suspension (3 × 10^9^ particles/ml).

Exosomes can also be characterized by the presence and enrichment of specific proteins as a consequence of their endosomal origin. Exosomes contain proteins involved in membrane transport and fusion (annexins, flotilin), in multivesicular bodies biogenesis (Tumor susceptibility gene 101; TSG101) and also integrins and tetraspanins (CD9, CD63, CD81; Thery et al., 2002) but will be devoid of proteins of mitochondrial, nuclear, ER, or Golgi origin. Our preparations were characterized by western blotting (Figure 1B) for the presence of exosomal markers Flotillin-1, CD9, and TSG101 and the absence of Golgi marker (GM130) or cytoskeleton marker (Actin) demonstrating the purity of our vesicles. Importantly, we were able to detect the presence of αsyn in our exosome preparation purified from SL1SL2 conditioned media when probing with anti-αsyn antibody (clone 4B12). To go further, the exosome pellet and supernatant were analyzed for luciferase activity, indicative of the presence of αsyn oligomeric species. As shown in Figure 1C, luciferase activity was detected in both the supernatant and the exosome samples. These data highlight the presence of two populations of αsyn oligomers in our experimental set up. The αsyn oligomers found in the supernatant are considered as “free” or “naked” αsyn, whereas αsyn oligomers in the exosome fraction are exosome-associated αsyn.

### Exosome-associated αsyn are efficiently internalized vs. free αsyn oligomers

**Figure 2:**
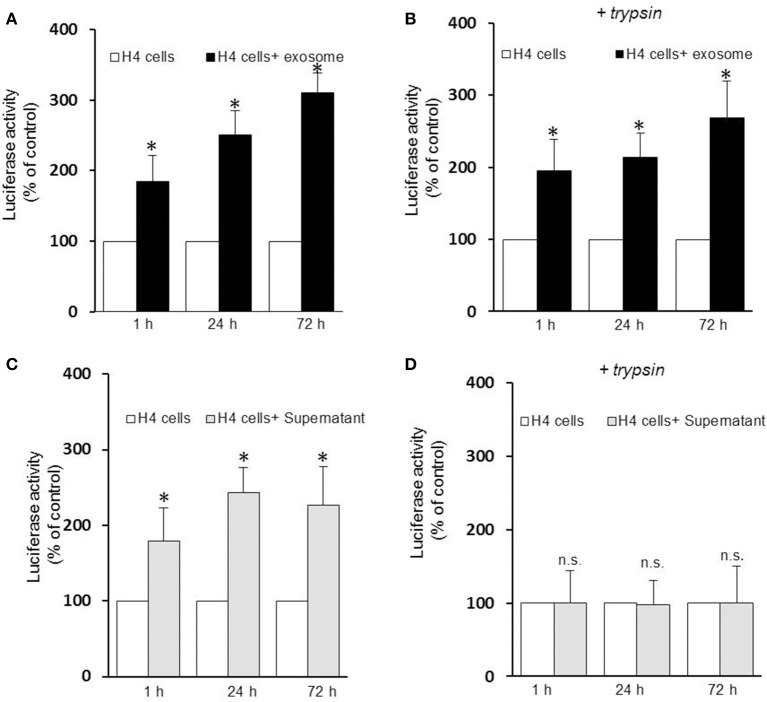
**Exosomes-associated αsyn oligomers are preferentially internalized by H_**4**_ cells**. Exosomes-associated αsyn oligomers bind to the cell membrane of recipient cell **(A)**, and get internalized **(B)** in a time dependent manner as seen by a significant increase of luciferase activity over time (*p < 0.05 as compared to control). **(C)** Free αsyn oligomers bind to the H4 recipient cells membrane but are not internalized **(D)**, trypisinization of the cell abolishes completely the luciferase signal observed previously (*p* > 0.0.05). Data are given as mean ± S.E.M., from three independent experiments. Statistical analysis was performed with one-way ANOVA, followed by Tukey HSD Multiple Comparison test; n.s, not significant.

Several lines of evidence suggest that exosomes are taken up by neighboring cells. To investigate the specific internalization of αsyn oligomers we set up an uptake assay using naïve H4 cells as recipient cells. Recipient cells were treated with either exosomes containing αsyn oligomers or free-αsyn oligomers from the supernatant and the subsequent entry into target cells was monitored using a luciferase assay at different time point. Luciferase activity was detected as early as 1 h after the addition of exosomes or the supernatant (Figures 2A,C) indicating rapid uptake of exosomes, and αsyn oligomers entered recipient cells in a time-dependent manner. Initially, no difference was observed in the internalization of free oligomers (supernatant) vs. exosome-associated oligomers suggestive of uptake that is independent of vesicle-association. However, to distinguish between internalized and surface-bound αsyn, we repeated the uptake assay including a brief trypsinization step before the luciferase assay. Interestingly, trypsinization of recipient cells had no effect on the luciferase activity from exosome treated cells (Figure 2B) but dramatically reduced the luciferase activity in cells treated with free αsyn oligomers to background levels (Figure 2D). These data demonstrate that whereas exosome-associated αsyn oligomers are internalized into recipient cells, free αsyn oligomers appear to remain bound to the extracellular cell surface and are not internalized.

### Effects of endocytosis inhibitors on internalization of αsyn-associated exosome

**Figure 3:**
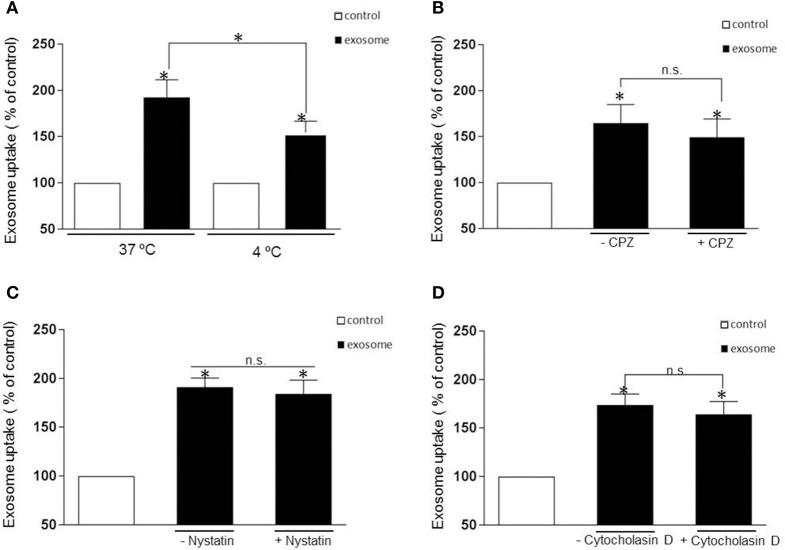
**Internalization is independent of clathrin- and caveolin-mediated endocytosis or macropynocytosis. (A)** Exosomes were added to recipient cells and incubate at 37°C or 4°C for 1 h. The temperature affects exosomal uptake with a significant decrease of luciferase activity at 4 degrees suggesting an energy-dependent process rather than passive membrane uptake (^*^p > 0.05). **(B–D)** Represent the % of internalization of exosomes with and without pharmacological treatment. The cells were pretreated with the following drug were used: CPZ 9 μg/ml (clathrin-dependent pathway inhibitor), nystatin 25 μg/ml (non-clathrin, caveolar-related pathway inhibitor) and Cytocholasin D 2 μM (macropinocytosis inhibitor) and add again with the exosomes. The luciferase signal was measured 1h later. None of the inhibitors significantly blocked the exosomal uptake (*P* > 0.05, n.s). Data were obtained from 3 independent experiments and are expressed as the % uptake relative to the control recipient cell. Values are the mean ± S.E.M.

The uptake mechanism of exosomes has been a matter of debate with the mechanism of entry of these nano vesicles still poorly understood. Multiple pathways that can mediate endocytosis, including phagocytosis, macropinocytosis, clathrin-mediated endocytosis, caveolae-mediated endocytosis have been hypothesized as possible routes of entry depending on the nature of the recipient cells. First, to rule out if exosomes containing αsyn oligomers enter cells through direct fusion with the plasma membrane of recipient cells (non-energy dependent process) or via endocytosis, we investigated the uptake efficiency at 37 or 4°C for 1 h incubation respectively. In this context we observed that incubation at 4°C efficiently attenuated significantly the uptake (Figure 3A), suggesting an energy-dependent process rather than passive membrane passage and consistent with an endocytic process rather than membrane fusion as exosomes followed a time (Figures 2A,B) and temperature-dependent pathway (Figure 3A).

Most experimental evidence suggests that EVs are taken up into endosomal compartments via endocytosis (Mulcahy et al., 2014). To further study the mechanism of αsyn oligomer internalization we next sought to define the distinct, cellular pathways associated with the endocytotic uptake of αsyn oligomers exosomes. To this end, we use specific pharmacological inhibitors chlorpromazine (CPZ) and nystatin to address the potential role of clathrin- and caveolin-mediated endocytosis, respectively. Before applying inhibiting treatments to study the uptake pathway, several control experiments were carried out. The efficacy of endocytosis inhibitors is cell type dependent and therefore controls of endocytosis inhibition were performed on H4 cells to test the activity of the treatments. Following addition of inhibitors we used fluorescent microscopy to evaluate the internalization of fluorescently labeled endocytic markers, transferrin (Tfn), and cholera toxin B (CTB), which are known to be specifically internalized by clathrin- and caveolin-mediated endocytosis respectively. Drug concentrations were optimized and conditions chosen such that the uptake of the relevant control substance was completely inhibited with no impaired cell morphology observed (Supplementary Figures 1A,C). To inhibit clathrin-mediated endocytosis, H4 cells were treated with CPZ at 9 μg/mL for 30 min prior to the addition of exosomes. This treatment completely blocked the endocytosis of Tfn (Supplementary Figure 1A) but did not significantly inhibit the entry of the exosomes (Figure 3B). Next, H4 cells were preincubated with 25 ug/ml nystatin before exposure to exosomes. Surprisingly, as with CPZ treatment, nystatin had no significant effect on the exosomal uptake (Figure 3C).

Another major endocytosis pathway, macropinocytosis, was then considered in our experimental procedure and we tested the macropinosome inhibitor, cytochalasin D at 2 μM. Once again there was no significant inhibitory effect on the internalization of the exosomes (Figure 3D) despite cytochalasin efficiently inhibiting the uptake of the specific fluid phase marker, Dextran D (Supplementary Figure 1B). Taken together, none of the inhibitors tested in this present study had a significant inhibitory effect on the internalization of αsyn containing exosomes.

### HSPGs does not mediate αsyn-exosomes uptake

**Figure 4:**
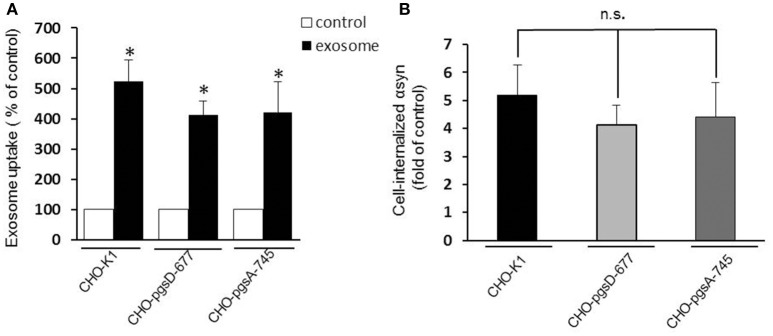
**HSPGs inhibition do not block αsyn oligomers uptake**. CHO-K1 (wild-type), CHO-pgsD-677, or CHO-pgsA-745 cells were incubated with exosomes for 1 h at 37°C. Internalization of αsyn was analyzed by luciferase assay. **(A)** The three types of CHO cells used as recipient cells were able to significantly internalize αsyn oligomers (^*^P > 0.05) after 1 h, and no significant differences could be observed in the uptake when comparing the wild type cells (CHO-K1) to the cells genetically modified for HSPGs (CHO- pgsD-677 and pgsA-745) **(B)**. Data are given as mean ± S.E.M., from 3 independent experiments. Statistical analysis: one-way ANOVA followed by Tuckey's multiple comparison test. n.s, not significant.

Heparan sulfate proteoglycans (HSPGs) are transmembrane and lipid-anchored cell surface receptors that interact with a variety of ligands triggering internalization. Previous studies have found a crucial role for HSPGs in selectively binding and internalizing exosomes in the cancer field (Christianson et al., 2013) and in internalizing infectious prion protein, aggregated tau, or Aβ monomer (Horonchik et al., 2005; Kanekiyo et al., 2011). Moreover Holmes et al. (2013) observed a clear colocalization of αsyn with HSPGs and found that they mediated the internalization of recombinant αsyn fibrils *in vitro*. Thus, we speculated that HSPGs might potentially mediate cellular binding and internalization of oligomeric αsyn-associated exosomes and that these anionic proteoglycans on the cell surface may serve as binding sites for the exosomes. To test the hypothesis, we performed an uptake assay using proteoglycan- deficient Chinese hamster ovary (PGD-CHO) cell lines as recipient cells. These cell lines have been widely characterized (Esko et al., 1985; Broekelmann et al., 2005) and were successfully used to demonstrate the critical role of HSPG in cellular Aβ binding and uptake (Kanekiyo et al., 2011). We used cells line lacking xylosyltransferase, an enzyme critical for glycosaminoglycan synthesis (pgsA-745), and the pgsD-677 cells deficient in *N*-acetylglucosaminyltransferase/glucuronyltransferase and compared exosome internalization to the wild-type control cell line, CHO-K1. Exosomes were added to the mutant and wild-type CHO cells as previously described and the internalization was assessed by monitoring intracellular luciferase activity. Surprisingly, we found exosomes containing αsyn oligomers were taken up equally well by cells that lack HSPGs as cells with HSPGs (CHO-K1) (Figures 4A,B), suggesting these molecules are not necessary for exosome endocytosis and may not be critical mediators of αsyn oligomer internalization.

## Discussion

EVs, and in particular exosomes, appear to play an important role in several physiological and pathological processes. They are seen as delivery machines capable of traveling between cells and unloading their contents across cell membranes mediating inter-cellular transfer. Several cell types can release these nanoparticles in the extracellular space and a number of aggregated proteins involved in neurodegenerative disease have been associated with EVs. A body of evidence suggests that exosomes may be involved in the spreading of misfolded neurodegenerative disease-associated proteins such as Prp, Tau, Aβ, and αsyn, and may be efficient initiators of disease propagation (Bellingham et al., 2012; Schneider and Simons, 2013). Recent studies have shown that exosomal αsyn can be detected in human cerebrospinal fluids (CSF; Stuendl et al., 2016) and blood plasma from PD patients (Shi et al., 2014) further emphasizing the importance of these nanovesicles in αsyn spreading mechanism. However, the exosome–cell interaction mode and their intracellular trafficking pathway in recipient cells remains incompletely understood. In this study, exosome internalization associated with αsyn oligomers was examined in a cellular assay. As previously reported by our group we confirmed oligomeric species of αsyn are detected in both the exosomal pellet and the exosome-free supernatant from the conditioned media (Danzer et al., 2012). Using an *in vitro* uptake assay combined with luciferase activity assay we provide direct evidence that free-αsyn oligomers cannot be internalized efficiently into recipient cells but only associate with the external surface of plasma membranes, as they could be removed by simple trypsinization. In contrast, trypsinization had no effect on exosome-treated cells because exosomes were readily internalized inside recipient cells. Our observations imply that although oligomeric αsyn occur in the extracellular milieu independent of exosomes, a small fraction of extracellular αsyn is released within EVs and is more prone internalization by recipient cells.

One of the central issues associated with secreted αsyn is to understand which species of αsyn are internalized by recipient cells that will eventually initiate intracellular toxic effects. In our hands, secreted vesicular αsyn appears to be readily internalized by neuroglioma cells compared to free αsyn oligomers and we and others have previously shown that exosomal αsyn confers toxicity on neighboring cells (Emmanouilidou et al., 2010; Danzer et al., 2012). By utilizing a protein complementation assay we directly track soluble αsyn oligomers and monitor αsyn aggregation with a surrogate luciferase readout. Therefore, we cannot exclude the possibility that free monomeric αsyn or free high molecular weight species of αsyn are efficiently internalized by neuroglioma recipient cells. Although in a parallel experiment (not shown) we performed an ELISA assay and still failed to detect internalization of αsyn in recipient cells indicating that free monomeric species were not taken up. In support of our data recent evidence suggests that αsyn uptake by neuronal cells (Luk et al., 2009) depends on the fibrilarization state of αsyn and cationic liposomes have been shown to be necessary for the internalization of recombinant fibrillar αsyn species into neuronal cells. Furthermore, Lee et al. (2008) suggested that aggregated αsyn is internalized more efficiently and via a different pathway, compared to monomeric αsyn protein. They suggest that monomeric αsyn directly translocate through the plasma membrane, which we were unable to measure with our complementation assay. It also appears that oligomers of αsyn exert greater cytotoxicity in recipient cells than soluble monomers (Desplats et al., 2009; Emmanouilidou et al., 2010), however, these data should be interpreted with caution, because they are based on high amounts of recombinant protein. Of note, our experimental setup used only cell-produced αsyn oligomers.

As described earlier, more free αsyn oligomers are detected in the preparation than exosome-associated. Although, the origin of free αsyn oligomers is still unclear it could be explained by extracellular degradation of the exosomal membrane by proteases or lipases allowing the release of proteins from the exosomal lumen to the extracellular matrix (Hughes, 1999). Nevertheless, in support of our observation only a small fraction of Aβ peptide is found associated with exosomes (Rajendran et al., 2006), and EV-associated proteins have been found in amyloid plaques of post mortem human brains (Thompson et al., 2016). In addition, Grey et al. showed that the propensity of αsyn to aggregate is increased by the presence of exosomes (Grey et al., 2015). Thus, one could speculate that exosomes may provide a favorable environment for the oligomerization process. In fact it has been shown that lipid-mediated oligomerization seems to be important in amyloid formation and polyunsaturated fatty acids have been shown to trigger multimerization of recombinant αsyn (Perrin et al., 2001). Interestingly when exosomes were isolated from CSF of PD patients, Stuendl et al. reported that the EVs were able to induce αsyn aggregation in a naïve cell line (Stuendl et al., 2016). The authors speculate that pathogenic αsyn oligomers may be preferentially sorted into exosomes and act as a seed into recipient cells. It is tempting to believe that exosomes may carry a pathogenic from of αsyn however, further in depth studies will be needed to determine if this is the case.

Since cell-to-cell propagation of pathogenic proteins has been deemed important in PD and neurodegenerative disorders, it is of interest to understand the pathway by which exosomes enter into cells. Most experimental evidence suggests that EVs are usually taken up via endocytosis (Mulcahy et al., 2014) but yet there appears to be little agreement in the literature as to which type of endocytic mechanisms are most important, with clathrin-dependent, caveolae dependent (Svensson et al., 2013), macropinocytosis (Fitzner et al., 2011; Tian et al., 2014), phagocytosis lipid raft-mediated uptake (Feng et al., 2010) or direct fusion with plasma membrane being the current postulates. Exosomal uptake is extremely rapid. We identified exosomes inside cells as early as 30 min after exposure. Our data also demonstrate that incubation at 4°C significantly reduces αsyn oligomer internalization, suggesting that uptake is an energy-requiring process as previously described (Christianson et al., 2013; Mulcahy et al., 2014; Tian et al., 2014). However, 30% of our exosomal population still gained access into the cells despite the low temperature. We therefore cannot neglect the fact that direct fusion with the plasma membrane may occur. Also, macropinocytosis-, clathrin-, and caveolin-dependent endocytosis inhibitors had no significant effect on exosome internalization in our assay. In a control experiment (not shown), exosomes isolated from cells in the presence of tetracycline and therefore not overexpressing αsyn underwent internalization that could not be blocked by cytochalasin D or nystatin suggesting that the presence of αsyn oligomers in exosomes does not contribute to the lack of inhibition. These differences might reflect the heterogeneity both in EV populations and in the cell types being used in the literature. The context of experiments may also affect the outcome and account for the observed discrepancies and different entry routes might reflect cell specialization or conditions. We can also speculate that multiple entry routes might even coexist in the same cell. Indeed any of the common endocytosis inhibitors used in our study abrogate exosome internalization suggesting that they may simultaneously trigger a number of different gateways and that different mechanisms are involved in internalization of recipient cell. Further experiments with tools such as antibodies to prevent receptor ligand interactions or RNAi may be of help to elucidate the endocytosis mechanisms involved.

Lastly various complexes, including viral particles and lipoproteins, use HSPGs to help gain entry into cells. Recently, cancer cell exosomes have been shown to depend on cell-surface HSPGs for their internalization (Christianson et al., 2013; Franzen et al., 2014). Interestingly, treatment of EVs with heparinase to remove surface proteoglycans had no effect on uptake, suggesting that it is the presence of HSPGs on the cell surface that are important for mediating vesicular entry (Christianson et al., 2013). Furthermore, Holmes et al. (2013) suggested that HSPGs mediate the uptake and seeding of αsyn recombinant fibrils. Even though evidence tends to suggest a crucial role of HSPGs in internalization of exosomes we could not observe this effect in our assay as cells genetically modified to be depleted of proteoglycans were still able to internalize exosome-associated αsyn. It is important to note that in the Holmes et al. study (Holmes et al., 2013) recombinant fibrillar proteins were used, which may explain the different results. Also, this result strongly correlates with our hypothesis that exosomes may gain entry into a cell via more than one route.

Further advancement of our understanding of both the EV uptake mechanism and interaction of αsyn with EVs are crucial and will enable development of therapeutic strategies for PD and others neurodegenerative disorders. Preventing the release of exosomes may become a novel and attractive approach to stop the spreading of oligomers of αsyn.

## Author contributions

MD carried out the study design, analysis, and drafting of the manuscript. TT isolated the exosomes and carried out the cellular uptake assay. AB and DM assisted with cell maintenance and isolation of the exosomes. SM provided the stable cell line and participated in the drafting and editing of the manuscript. IY and TP carried out the nanosight reading. PM participated in the experimental design, coordination, interpretation, drafting and editing of the manuscript. All authors read and approved the final manuscript.

### Conflict of interest statement

The authors declare that the research was conducted in the absence of any commercial or financial relationships that could be construed as a potential conflict of interest.

## Supplementary material

Supplementary Figure 1 **Internalization of control fluorescent markers for clathrin- and caveolin mediated endocytosis or macropynocytosis**. Fluorescence images represent internalization of **(A)** Alexa Fluor 488-Transferrin (Tfn), **(B)** Alexa Fluor 488-Dextran D, and **(C)** Alexa Fluor 488-Cholera Toxin B (CTB) in H4 cells with and without drug treatment. CPZ (clathrin-dependent pathway inhibitor), Cytochalsain D (macropinocytosis) and nystatin (non-clathrin, caveolar-related pathway inhibitor) were able to significantly decrease the internalization of their proper marker at the given concentration. Scale bars, 15 μm.
