# Supplementary figures and images for: Investigation of Endocytic Pathways for the Internalization of Exosome-Associated Oligomeric Alpha-Synuclein
Source: Front Neurosci. 2017 Mar 30;11:172. doi: 10.3389/fnins.2017.00172 (PMC5371652; doi:10.3389/fnins.2017.00172)

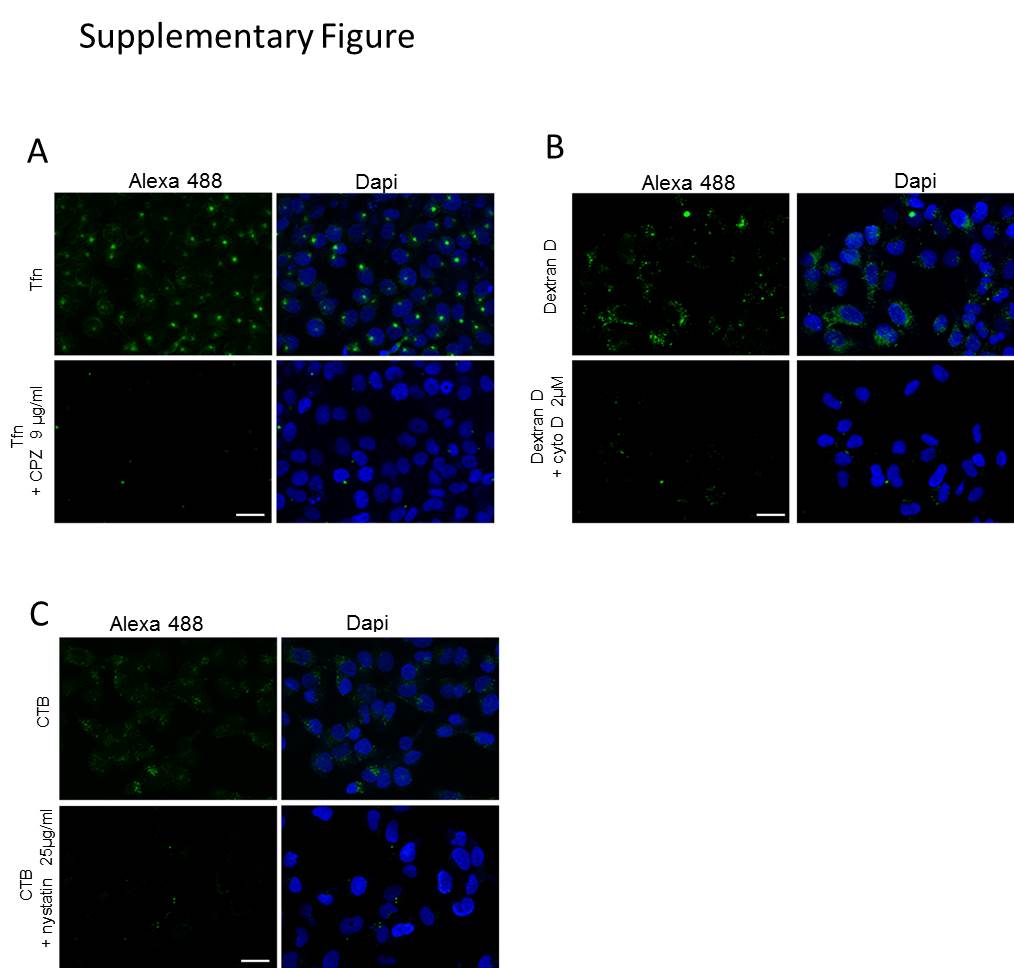

Supplement: Supplementary Figure 1 — Internalization of control fluorescent markers for clathrin- and caveolin mediated endocytosis or macropynocytosis. Fluorescence images represent internalization of (A) Alexa Fluor 488-Transferrin (Tfn), (B) Alexa Fluor 488-Dextran D, and (C) Alexa Fluor 488-Cholera Toxin B (CTB) in H4 cells with and without drug treatment. CPZ (clathrin-dependent pathway inhibitor), Cytochalsain D (macropinocytosis) and nystatin (non-clathrin, caveolar-related pathway inhibitor) were able to significantly decrease the internalization of their proper marker at the given concentration. Scale bars, 15 μm. [file Image1.JPEG]
